# Whole Genome Sequencing of Pediatric *Klebsiella pneumoniae* Strains Reveals Important Insights Into Their Virulence-Associated Traits

**DOI:** 10.3389/fmicb.2021.711577

**Published:** 2021-08-13

**Authors:** Mauricio Flores-Valdez, Miguel A. Ares, Roberto Rosales-Reyes, Javier Torres, Jorge A. Girón, Bart C. Weimer, Alfonso Mendez-Tenorio, Miguel A. De la Cruz

**Affiliations:** ^1^Unidad de Investigación Médica en Enfermedades Infecciosas y Parasitarias, Hospital de Pediatría, Centro Médico Nacional Siglo XXI, Instituto Mexicano del Seguro Social, Mexico City, Mexico; ^2^Laboratorio de Biotecnología y Bioinformática Genómica, Escuela Nacional De Ciencias Biológicas (ENCB), Instituto Politécnico Nacional, Mexico City, Mexico; ^3^Departamento de Microbiología, Escuela Nacional de Ciencias Biológicas, Instituto Politécnico Nacional, Mexico City, Mexico; ^4^Unidad de Medicina Experimental, Facultad de Medicina, Universidad Nacional Autónoma de México, Mexico City, Mexico; ^5^Centro de Detección Biomolecular, Benemérita Universidad Autónoma de Puebla, Puebla, Mexico; ^6^Department of Population Health and Reproduction, School of Veterinary Medicine, 100K Pathogen Genome Project, University of California, Davis, Davis, CA, United States

**Keywords:** *Klebsiella pneumoniae*, whole-genome sequence, Mexico, blood samples, phylogenomic

## Abstract

*Klebsiella pneumoniae* is recognized as a common cause of nosocomial infections and outbreaks causing pneumonia, septicemia, and urinary tract infections. This opportunistic bacterium shows an increasing acquisition of antibiotic-resistance genes, which complicates treatment of infections. Hence, fast reliable strain typing methods are paramount for the study of this opportunistic pathogen’s multi-drug resistance genetic profiles. In this study, thirty-eight strains of *K. pneumoniae* isolated from the blood of pediatric patients were characterized by whole-genome sequencing and genomic clustering methods. Genes encoding β-lactamase were found in all the bacterial isolates, among which the *bla*_SHV_ variant was the most prevalent (53%). Moreover, genes encoding virulence factors such as fimbriae, capsule, outer membrane proteins, T4SS and siderophores were investigated. Additionally, a multi-locus sequence typing (MLST) analysis revealed 24 distinct sequence types identified within the isolates, among which the most frequently represented were ST76 (16%) and ST70 (11%). Based on LPS structure, serotypes O1 and O3 were the most prevalent, accounting for approximately 63% of all infections. The virulence capsular types K10, K136, and K2 were present in 16, 13, and 8% of the isolates, respectively. Phylogenomic analysis based on virtual genome fingerprints correlated with the MLST data. The phylogenomic reconstruction also denoted association between strains with a higher abundance of virulence genes and virulent serotypes compared to strains that do not possess these traits. This study highlights the value of whole-genomic sequencing in the surveillance of virulence attributes among clinical *K. pneumoniae* strains.

## Introduction

*Klebsiella pneumoniae* is a ubiquitous Gram-negative encapsulated bacterium that is commonly found in the mucosal surfaces of mammals and the environment. In humans, these bacteria can colonize the gastrointestinal and nasopharynx tracts, from where they gain entry to blood circulation and other tissues causing infection (Piperaki et al., 2017). *K. pneumoniae* is well-known as one of the major causes of community- and hospital-acquired Gram-negative bloodstream infections (Meatherall et al., 2009). In immunocompromised patients it can cause biliary infection, peritonitis, meningitis, septicemia, and liver abscesses (Ares et al., 2013). Also, *K. pneumoniae* nosocomial isolates often display high rates of antimicrobial multi-drug resistance (Chong et al., 2018). This opportunistic microorganism causes infection through several virulence factors such as surface antigens, fimbriae, capsule, outer membrane proteins, and siderophores; all of which allow the bacteria to enter and multiply within the host (Podschun and Ullmann, 1998; Ares et al., 2016, 2019). The genus *Klebsiella* expresses two types of surface antigens: a lipopolysaccharide (O antigen) and a capsular polysaccharide (K antigen). The *K. pneumoniae* capsule is a complex structure that measures up to 160 nm wide (Clements et al., 2008). The number of genes that code for the capsule of *K. pneumoniae* vary from 16 to 20 genes according to the capsular serotype. The capsular polysaccharide synthesis (*cps*) locus comprises genes that are organized into three consecutive transcriptional units that give rise to a large number of proteins involved in biosynthesis and export of capsular components. The capsule is the most thoroughly studied virulence factor of *K. pneumoniae*. The CPS confers resistance against the bactericidal activity of antimicrobial peptides, complement, and phagocytes (Paczosa and Mecsas, 2016). In addition, the capsule also protects the bacterium from opsonization and suppresses the early inflammatory response (Ko, 2017; Ares et al., 2019). The LPS is essential for the bacterial cell envelope structure, protects against antibiotics, and promotes adhesion. The LPS consists of three domains: the lipid A, an oligosaccharide core and the O antigen. The polysaccharide chains of the O antigen facilitate the initial adhesion process and confer resistance to serum bactericidal activity (Podschun and Ullmann, 1998; Stojkovic et al., 2017). Thus, both capsule and LPS antigens significantly contribute to the pathogenicity of the bacteria (Evrard et al., 2010). Seventy-eight K antigens and eight O antigens have been described in *K. pneumoniae* strains. The structural variability of these antigens has been used to classify strains into several serotypes, which in turn have elucidated differences between the degree of virulence among different strains (Follador et al., 2016). Strains carrying the K1 and K2 capsular serotypes, which mainly cause liver abscess, are known to be hypermucoviscous (Chung et al., 2012) and the serotype O1 are the most prevalent in clinical infections (Hsieh et al., 2014). Host cell adherence and biofilm formation of *K. pneumoniae* isolates are mediated by type 1, type 3, and ECP fimbriae whose major fimbrial subunits are encoded by the *fimA*, *mrkA*, and *ecpA* genes, respectively (Alcántar-Curiel et al., 2013). OmpA is one of the major outer membrane proteins of Gram-negative bacteria, and it is highly conserved among the *Enterobacteriaceae*. *K. pneumoniae*’s OmpA contributes to the attenuation of the airway epithelial cell-mediated inflammatory response and the absence of OmpA makes the microorganism more susceptible to antimicrobial peptides (Li et al., 2014). Additionally, *K. pneumoniae* produces two major porins OmpK35 and OmpK36, which are homologs of OmpF and OmpC of *Escherichia coli*, respectively, functioning as channels for export and import of hydrophilic molecules (e.g., nutrients and cephalosporins/carbapenems). The loss of outer membrane porins in *K. pneumoniae* leads to increased resistance to cephalosporins/carbapenems and reduced virulence (March et al., 2013; Li et al., 2014). The production of siderophores confers to bacteria the ability to sequester and uptake iron from infected tissues. *K. pneumoniae* produces different siderophores among which the most relevant are enterobactin, yersiniabactin, aerobactin, and salmochelin. In this study, we characterized the most relevant phenotypic and genomic virulence traits occurring among a set of 38 clinical *K. pneumoniae* strains isolated from a pediatric population in Mexico City. The outcome of this study highlights the importance whole genome sequence analysis in the surveillance of virulence attributes of clinical *K. pneumoniae* strains.

## Materials and Methods

### Clinical Isolates

*Klebsiella pneumoniae* strains were isolated (September 2010 to August 2013) from individuals diagnosed with sepsis from the Laboratory of Clinical Microbiology, Pediatric Hospital, Centro Médico Nacional Siglo XXI, Instituto Mexicano del Seguro Social (IMSS), Mexico City, Mexico (Ares et al., 2013). All pediatric patients included in this study were hospitalized for more than 48 h. A clinical examination was conducted by the medical staff in order to rule out sepsis acquired outside the hospital. Microbiological methods were performed as described (Ares et al., 2013). To isolate and identify the microbial species, positive blood cultures were selected. An aliquot of 50 μL was inoculated onto MacConkey agar plates and incubated in aerobic conditions for 24–48 h at 37°C. After incubation, colonies grown on agar plates were suspended in 3 mL of sterile saline solution (0.45% NaCl, pH 7.0) to reach a McFarland turbidity of 0.5–0.63. Bacterial identification was carried out using the VITEK-2 system (bioMérieux) and genomic DNA was extracted using the DNeasy Mini Kit (Qiagen, Hilden, Germany). The clinical data are shown in Table 1.

**TABLE 1:** Clinical data of *K. pneumoniae* strains.

**Strain**	**Clinical service**	**Age**	**Gender**	**Date**	**Diagnosis**
2k	NICU	Newborn	M	September-10	Sepsis
4k	PREESCHOOL CHILDREN	3 years	M	September-10	Fibroblastoma
5k	CHILDREN/ADOLESCENT	7 years	M	October-10	Sepsis
6k	PITU	10 years	F	November-10	Sepsis
7k	INFANTS	10 months	M	November-10	Sepsis
8k	NICU	Newborn	M	Dic-10	Sepsis
9k	PREESCHOOL CHILDREN	2 years	F	February-11	Sepsis
10k	NICU	Newborn	M	March-11	Sepsis
11k	CHILDREN/ADOLESCENT	8 years	M	June-11	Osteosarcoma
12k	PREESCHOOL CHILDREN	3 years	M	May-11	Sepsis
13k	INFANTS	3 months	M	May-11	Sepsis
15k	NICU	Newborn	F	July-11	Sepsis
16k	NICU	Newborn	F	September-11	Sepsis
17k	PREESCHOOL CHILDREN	4 years	M	October-11	Pneumonia
18k	INFANTS	6 months	M	November-11	Sepsis
19k	INFANTS	5 months	M	December-11	Sepsis
20k	CHILDREN/ADOLESCENT	8 years	F	January-12	Sepsis
21k	INFANTS	4 months	M	January-12	Sepsis
22k	NICU	Newborn	F	February-12	Sepsis
23k	NICU	Newborn	F	February-12	Sepsis
24k	INFANTS	5 months	M	May-12	Sepsis
25k	NICU	Newborn	F	June-12	Sepsis
27k	NICU	Newborn	F	July-12	Sepsis
28k	NICU	Newborn	M	August-12	Sepsis
30k	NICU	Newborn	M	September-12	Sepsis
31k	NICU	Newborn	M	September-12	Sepsis
34k	NICU	Newborn	F	October-12	Sepsis
35k	NICU	Newborn	F	October-12	Sepsis
36k	PREESCHOOL CHILDREN	5 years	F	November-12	Sepsis
37k	PREESCHOOL CHILDREN	4 years	M	November-12	Sepsis
40k	INFANTS	3 months	M	November-12	Sepsis
42k	PREESCHOOL CHILDREN	2 years	M	Dicr-12	Sepsis
43k	NICU	Newborn	M	Dic-12	Sepsis
45k	NICU	Newborn	F	January-13	Sepsis
46k	CHILDREN/ADOLESCENT	7 years	M	February-13	Sepsis
48k	NICU	Newborn	F	May-13	Sepsis
49k	NICU	Newborn	M	July-13	Sepsis
50k	NICU	Newborn	M	August-13	Sepsis

### Antimicrobial Susceptibility Testing

Antimicrobial susceptibility testing for *K. pneumoniae* strains was carried out using Gram-negative bacilli cards (VITEK-2, AST- GN04) as was previously reported (Ares et al., 2013).

### Whole-Genome Sequencing

Genomic DNA was obtained from 50 random strains of the clinical *K. pneumoniae* strain collection and subjected to whole genome sequencing with the HiSeq 2000 platform (Illumina) producing 150 bp paired end reads at the laboratory of Dr. Bart Weimer, University of California, Davis. *De novo* genome assembly was accomplished using SPAdes v.3.13.0 (Bankevich et al., 2012) using a range of *k*-mer sizes of 21, 33, 55, 77-mers for the assembly. The assembled draft genomes were annotated by Prokka v.1.13 (Seemann, 2014). Finally, 38 *K. pneumoniae* genomes showed good data quality and were deposited at NCBI. Five *Klebsiella quasipneumoniae* (29k, 33k, 39k, 41k, and 44k) and three *Klebsiella variicola* (3k, 26k, and 32k) strains were added to the bioproject. In addition, strain 1k, which is the genome of wild-type *K. pneumoniae* used in our group, was also included (Ares et al., 2016).

### Data Access

Sequence data were submitted to the NCBI’s Gen Bank under BioProject Accession Number PRJNA605025. Each of the strains is stored with the Accession Numbers SAMN14008666-SAMN14008712 and the genomic sequences can be accessed with the Accession Numbers JAAGZT000000000-JAAHBN000000000.

### Phylogenomic Analysis

Genome sequences of both *K. pneumoniae* isolates and NTUH K-2044 reference strain (Wu et al., 2009) were provided in FASTA format to calculate a phylogenomic tree based on Virtual Genome Fingerprints (VGF) using the VAMPhyRE software^1^. This method consists of carrying out a virtual hybridization analysis in which the recognition sites of a collection of 15,264 13-mer probe sequences previously designed to maximize their diversity, are searched in both strands of each genome and allowing a single-base mismatch during hybridization. The collection of hybridization sites obtained by this technique is known as the Virtual Genomic Fingerprint (VGF) and consists of a sample of sites regularly distributed throughout the genome. The VGFs are compared pairwise to estimate the fraction of shared homologous sites. A site comparison optimization analysis is previously performed, including a symmetric extension to both sites of genome alignment at each hybridization site and applying threshold match value that guarantees that only those sites not shared by chance are counted. From the fraction of homologous sites shared between each pair of genomes, a measure of their genomic distance, equivalent to the number of nucleotide substitutions between each pair, is estimated according to a method described by Nei and Li (1979). The distance is used in the MEGAX program to calculate phylogenetic trees using the Neighbor-Joining method (Kumar et al., 2018). Phylogenomic trees were edited with iTol (Interactive Tree of Life). Likewise, all assembled genomes in the dataset study were annotated using the Prokka prokaryotic annotation pipeline (Seemann, 2014) and the pan-genome was calculated with Roary: The Pan Genome Pipeline (Page et al., 2015) negative bacilli cards (VITEK-2, AST- GN04), as was previously reported (Ares et al., 2013).

### Multilocus Sequence Typing

Multilocus sequence typing (MLST) analysis was performed using seven housekeeping genes (*gapA, infB, mdh, pgi, phoE, rpoB*, and *tonB*) (Diancourt et al., 2005). The allele sequences and sequence types (STs) were determined by the BIGS database of the Institute Pasteur^2^ (Jolley and Maiden, 2010). Only exact matches of the allelic profile were reported.

### Antibiotic Resistance

The bacterial resistome was obtained by assessing the occurrence of antimicrobial resistance genes and plasmid replicons using both ResFinder 3.1 (Zankari et al., 2012) and PlasmidFinder 2.0 (Carattoli et al., 2014) from the Center for Genomic Epidemiology^3^. The analysis was performed with an identity threshold of 98% and a minimum match length of 80%. The families of the antibiotics assessed were fosfomycin, quinolones, phenicoles, aminoglycosides, sulphonamides, trimethoprim, tetracycline, and β-lactam.

### Virulence Factors

Known virulence genes were searched on the *K. pneumoniae* genomes obtained above with BLASTN (Altschul et al., 1990; Camacho et al., 2009). For type 1 and type 3 fimbriae the clusters *fim* (*fimB, fimE, fimA, fimI, fimC, fimD, fimF, fimG, fimH*, and *fimK*) and *mrk* (*mrkA, mrkB, mrkC, mrkD*, and *mrkF*) were used, respectively. For ECP, the *ecpA*, *ecpB*, *ecpC*, *ecpD*, and *ecpE* gene cluster was analyzed. Likewise, the genes that code for outer membrane proteins (*ompA*, *ompK35*, and *ompK36*), the cluster *virB* (*virB1, virB2, virB3-B4, virB5, virB6, virB7, virB8, virB9, virB10*, and *virB11*) of the T4SS, and the hypermucoviscous associated genes *magA* and *rmpA* were screened. Additionally, *fes, iroN, fyuA*, and *iutA* genes, which correspond to enterobactin, salmochelin, yersiniabactin, and aerobactin siderophores respectively, were also searched. All the genes used for querying were obtained from the NTUH K-2044 reference strain. All analyses were carried out with an identity threshold of 96% and a minimum match length of 80%.

### Serotyping (K and O Antigens)

The genes *wzi* and *wzc* were searched in the draft genomes of *K. pneumoniae* using BLASTN (Altschul et al., 1990; Camacho et al., 2009), a database was assembled utilizing the corresponding locus of K serotypes published by Wyres et al. (2016). In the same manner the locus *wb* was searched and compared with the data published by Fang et al. (2016) to identify O serotypes. All analyses were carried out with an identity threshold of 96% and a minimum length of 80%. Additionally, the software Kaptive^4^ (Wick et al., 2018) was used to determine the K and O serotypes. Results that matched in both methodologies were reported as positive for the searched serotype.

### Glucuronic Acid Analysis

Capsular polysaccharides were extracted and quantified as previously described (Ares et al., 2016). The glucuronic acid concentration in each bacterium was expressed in micrograms/10^9^ CFU.

### Phagocytosis of Bacteria by THP-1 Macrophages

The phagocytosis assay was performed using THP-1 (ATCC TIB-202) human monocytes as previously described (Ares et al., 2016). Phagocytosis resistance was expressed in intracellular colony-forming-units per mL (CFUs/mL).

### Antimicrobial Activity of Polymyxin B

All experiments were performed with an initial inoculum of ∼10^6^ CFU/mL in 20 mL of LB medium in 50 mL pyrogen-free and sterile polypropylene tubes. Polymyxin B activity was examined at 4 mg/mL. Serial strains in 20 mL were collected at 4 h for viable counting on LB agar plates after 24 h of incubation at 37°C as described Nang et al., 2019.

## Results

### Whole-Genome Sequencing of *K. pneumoniae* Strains

Genomic sequences of 38 bacterial strains positively identified as *K. pneumoniae* were obtained and they were named with a number followed by a letter “k” (e.g., 1k; Table 1). The draft genomes had an average assembly length of 5.55 Mb ± 0.14; a GC content of 57.23% ± 0.16%; N50 of 169,445 ± 41,243; and CDS of 5,135 ± 144 (assembly data and accession are shown in Table 2). The *K. pneumoniae* isolates shared a core genome consisting of 1,192 genes (present in ≥99% isolates and with 95% identity). The genomes analysis showed that with exception of strain 46k, all *K. pneumoniae* presented at least one plasmid sequence (Table 3).

**TABLE 2:** Assembly data of *K. pneumoniae* genomes.

**Strain**	**Contigs**	**Assembly length**	**(G + C)**	**N50**	**CDS**	**Accession code**
2k	260	5483120	57.13	174020	5017	JAAGZU
4k	222	5530083	57.13	125462	5123	JAAGZW
5k	320	5573325	57.31	115852	5122	JAAGZX
6k	340	5553521	57.31	153732	5096	JAAGZY
7k	245	5542493	57.28	129744	5159	JAAGZZ
8k	257	5594033	57.17	177899	5264	JAAHAA
9k	319	5530716	57.22	275308	5115	JAAHAB
10k	200	5390094	57.3	175415	4963	JAAHAC
11k	321	5614338	57.14	225004	5261	JAAHAD
12k	435	5525991	57.21	133159	5113	JAAHAE
13k	339	5748782	56.95	173047	5299	JAAHAF
15k	225	5394489	57.35	141238	5014	JAAHAG
16k	995	5713124	57.22	216331	5078	JAAHAH
17k	192	5387461	57.35	132433	5017	JAAHAI
18k	239	5433473	57.39	172351	5031	JAAHAJ
19k	293	5391084	57.42	186244	4978	JAAHAK
20k	377	5706753	57	174915	5237	JAAHAL
21k	387	5468419	57.3	162734	5030	JAAHAM
22k	1216	5957453	56.74	223553	5358	JAAHAN
23k	305	5492608	57.3	194443	5087	JAAHAO
24k	226	5368355	57.45	166439	4935	JAAHAP
25k	336	5687554	57.05	190938	5349	JAAHAQ
27k	249	5532662	57.18	127081	5178	JAAHAS
28k	445	5620676	57.22	93364	5228	JAAHAT
30k	255	5578383	57.27	180021	5169	JAAHAV
31k	354	5588722	57.22	105082	5219	JAAHAW
34k	183	5624505	57.23	193631	5235	JAAHAZ
35k	258	5643179	57.21	180051	5237	JAAHBA
36k	188	5500354	57.31	191536	5109	JAAHBB
37k	200	5505486	57.3	185559	5105	JAAHBC
40k	786	5559365	57.4	273751	5059	JAAHBE
42k	404	5678007	57.2	158811	5248	JAAHBG
43k	419	5609558	57.2	185313	5201	JAAHBH
45k	352	5572546	57.01	187574	5134	JAAHBJ
46k	246	5204737	57.61	86376	4763	JAAHBK
48k	220	5731896	57.05	172510	5344	JAAHBL
49k	304	5726980	57.14	129731	5359	JAAHBM
50k	420	5666464	57.19	159883	5259	JAAHBN

**TABLE 3:** Plasmid replicons found in *K. pneumoniae* strains.

**Strain**	**Plasmid replicons**
2k	IncFIA(HI1), IncFIB(K), IncHI1B, ColRNAI
4k	IncFIB(K), IncFII(K)
5k	IncFIB(K), IncFII(K)
6k	IncFIB(K), ColRNAI
7k	IncFIB(K), IncL/M(pMU407)
8k	IncN3, IncFIB(K)
9k	IncFIB(K), IncFII(K)
10k	IncFIB(K), IncFII(K)
11k	IncN3, IncFIB(K)
12k	IncFIA(HI1), IncFII, IncFIB(K), IncN
13k	IncFIB(pQil), IncFIB(K), IncFII(K)
15k	IncFII, IncFIB(K), Col(MGD2), IncR
16k	IncFII, IncFIB(K)
17k	IncFII, IncFIB(K), IncR
18k	IncFII, IncFIB(K), IncR
19k	IncFIB(K), IncFII(K)
20k	IncFIB(K), IncFII(K)
21k	IncFIA(HI1), IncFIB(K), IncR
22k	IncFIB(K), IncFII(K)
23k	IncFIB(K)
24k	IncFII, IncFIB(K)
25k	IncN3, IncFIB(K), IncFII(K)
27k	IncFIB(K), IncA/C2
28k	IncFIB(K), IncFII(K), IncFII(Y), Col(MGD2), Col(BS512), IncR, ColRNAI
30k	IncFIB(K), IncA/C2, ColRNAI
31k	IncFIB(K), IncFII(K), IncFII(Y), IncR
34k	IncFIB(K), IncA/C2
35k	IncFIB(K), IncA/C2
36k	IncFIB(K), Col(MGD2), IncA/C2
37k	IncFIB(K), IncA/C2
40k	IncFII, IncFIB(K)
42k	IncFIB(K), Col(MGD2), IncA/C2
43k	IncA/C2
45k	IncFIB(K)
46k	NA
48k	IncFIB(pKPHS1), IncFIB(K), IncR
49k	IncFIB(K), IncA/C2
50k	IncFIB(K), Col(MGD2), IncA/C2

### Virtual Genomic Fingerprinting (VGF) Correlates With MLST

**FIGURE 1:**
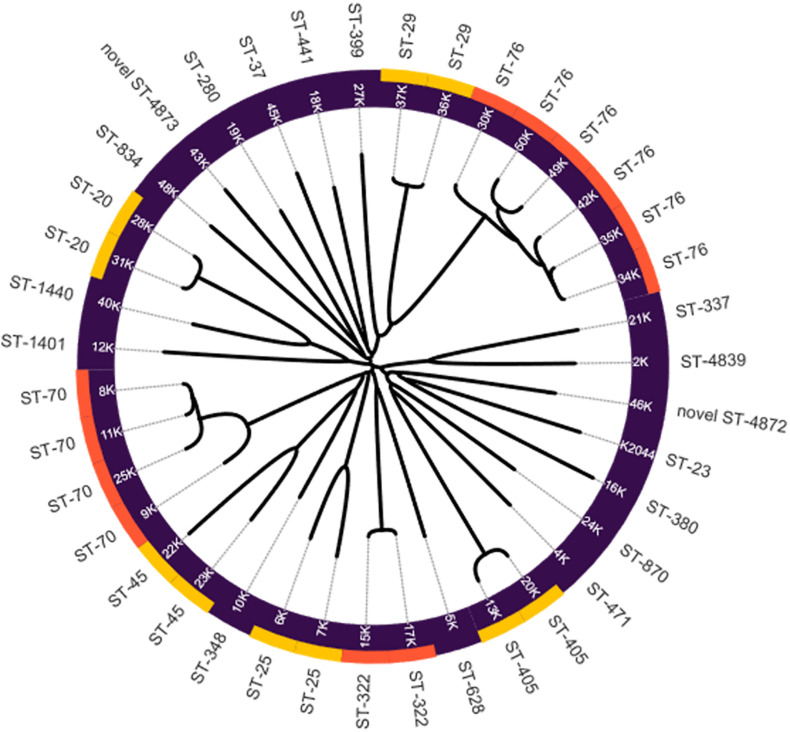
Phylogenomic reconstruction of *Klebsiella pneumoniae* strains by VGF. NTUH-K2044 strain was added as reference. Yellow and orange colors denote the clustering of strains by MLST.

Twenty-four different sequence types (ST) were identified among the *K. pneumoniae* isolates in this study. The most prevalent STs were ST-76 in six strains followed by ST-70 with four occurrences (Table 4). Interestingly, two *K. pneumoniae* strains, 46k and 43k, presented two new sequence types ST-4872 and ST-4873, respectively. Our analysis showed that ST-76 and ST-70 are the most prevalent ST in the strains collection and that new STs such as ST-4872 and ST-4873, appeared in the bacterial population of *K. pneumoniae*. The phylogenetic analysis was carried out based on the virtual genomic fingerprint technique (VAMPhyRE) using 13 k-mer probes and the Nearest-Neighbor method in MEGAX with default settings. The final phylogenomic representation (Figure 1) denotes consistent correlation with MLST findings.

**TABLE 4:** Capsular polysaccharide synthesis (CPS), LPS, and MLST in *K. pneumoniae* strains.

**Strain**	**LPS**	**CPS**	**MLST**
2k	O1	K28	ST-4839
4k	O2	K57	ST-471
5k	O3	K123	ST-628
6k	O1	K2	ST-25
7k	O1	K2	ST-25
8k	O1	K136	ST-70
9k	O1	K136	ST-70
10k	O1	K62	ST-348
11k	O1	K136	ST-70
12k	OL101	K49	ST-1401
13k	O4	K58	ST-405
15k	O4	K17	ST-322
16k	O1	K2	ST-380
17k	O4	K17	ST-322
18k	O2	K33	ST-441
19k	O2	K23	ST-280
20k	O4	K58	ST-405
21k	O1	K33	ST-337
22k	O2	K62	ST-45
23k	O2	K24	ST-45
24k	O1	K17	ST-870
25k	O1	K136	ST-70
27k	O2	K136	ST-399
28k	O1	K28	ST-20
30k	O3	K10	ST-76
31k	O1	K28	ST-20
34k	O3	K10	ST-76
35k	O3	K10	ST-76
36k	O1	K30	ST-29
37k	O1	K30	ST-29
40k	O2	K27	ST-1440
42k	O3	K10	ST-76
43k	O3	K24	ST-4873*
45k	O4	K15	ST-37
46k	O3	K39	ST-4872*
48k	O3	K123	ST-834
49k	O3	K10	ST-76
50k	O3	K10	ST-76

*means new MLST found in this study.

### Resistome of *K. pneumoniae* Strains

The results for the search of resistance genes in the *K. pneumoniae* strains studied is presented in Table 5. The prevalence of genes encoding resistance to fosfomycin, quinolones, phenicols, aminoglycosides, sulphonamides, trimethoprim, tetracycline was 100, 97, 5, 79, 68, 39, and 45%, respectively. Moreover, β-lactamase genes (Table 5) such as *bla*_SHV_, *bla*_OXA_, *bla*_TEM_, and *bla*_CTX–M_ were present in 53, 53, 5, and 26%, of the strains, respectively. No *bla*_OKP_, *bla*_LEN_ genes were found in this bacterial collection. All the strains carried at least two different types of resistance genes. Antimicrobial susceptibility testing of *K. pneumoniae* strains (Table 6) showed a similar profile correspondent to the resistance genes encoded in the bacterial genome.

**TABLE 5:** Antibiotic resistance genes in *K. pneumoniae* strains.

**Strain**	**Fosfomycin**	**Quinolones**	**Phenicols**	**Aminoglycosides**	**Sulphonamides**	**Trimethoprim**	**Tetracycline**	**b-lactam**
2k	*fosA*	*oqxA, qnrB*		*aac(3), aadA1, strB*	*sul1*	*dfrA*	*tet(A)*	*bla_SHV–__28_*
4k	*fosA*	*oqxA, qnrB*	*catB3*	*aac(3), strA, strB*	*sul2*	*dfrA*	*tet(A)*	*bla_CTX–M–__15_, bla_OXA–__1_, bla_TEM–__1__B_*
5k	*fosA*	*oqxA, qnrB*	*catB3*	*aac(3), strA, strB*	*sul2*	*dfrA*	*tet(A)*	*bla_CTX–M–__15_, bla_OXA–__1_, bla_SHV–__1_, bla_TEM–__1__B_*
6k	*fosA*	*oqxA, qnrB*	*catB3*	*aac(3), strA, strB*	*sul2*	*dfrA*	*tet(A)*	*bla_CTX–M–__15_, bla_OXA–__1_, bla_TEM–__1__B_*
7k	*fosA*	*oqxA*		*aac(3)*				*bla_CTX–M–__15_, bla_TEM–__1__B_*
8k	*fosA*	*oqxA*		*aac(6′)-33, aadA1*	*sul1*			*bla_OXA–__2_*
9k	*fosA*	*oqxA, qnrB*	*catB3*	*aac(3),strA, strB*	*sul2*	*dfrA*	*tet(A)*	*bla_CTX–M–__15_, bla_OXA–__1_, bla_TEM–__1__B_*
10k	*fosA*	*oqxA, qnrB*	*catB3*	*aac(3), strA, strB*	*sul2*	*dfrA*	*tet(A)*	*bla_CTX–M–15_, bla_OXA–1_, bla_TEM–1B_*
11k	*fosA*	*oqxA*		*aac(6′), aadA1*	*sul1*			*bla_OXA–__2_*
12k	*fosA*	*oqxA*						*bla_SHV–__12_*
13k	*fosA*	*oqxA, qnrB*	*catB3*	*aac(3), strA, strB*	*sul2*	*dfrA*	*tet(A)*	*bla_CTX–M–15_, bla_OXA–1_, bla_TEM–1B_*
15k	*fosA*	*oqxA*						*bla_SHV–__11_*
16k	*fosA*	*oqxA*						*bla_SHV–__1_*
17k	*fosA*	*oqxA*						*bla_SHV–__11_*
18k	*fosA*							*bla_SHV–__38_*
19k	*fosA*	*oqxA*,	*catB3*	*strA, strB*	*sul2*	*dfrA*	*tet(A)*	*bla_CTX–M–15_, bla_OXA–1_, bla_TEM–1B_*
20k	*fosA*	*oqxA, qnrB*	*catB3*	*aac(3), strA, strB*	*sul2*	*dfrA*	*tet(A)*	*bla_CTX–M–15_, bla_OXA–1_, bla_TEM–1B_*
21k	*fosA*	*oqxA*		*strA, strB*	*sul2*	*dfrA*	*tet(A)*	*bla_SHV–__11_*
22k	*fosA*	*oqxA, qnrB*		*strA, strB*	*sul2*	*dfrA*	*tet(A)*	*bla_CTX–M–15_, bla_SHV–1_, bla_TEM–1B_*
23k	*fosA*	*oqxA*					*tet(A)*	*bla_SHV–1_*
24k	*fosA*	*oqxA*		*strA, strB*			*tet(A)*	*bla_SHV–__1_*
25k	*fosA*	*oqxA*		*aac(6′), aadA1*	*sul1*			*bla_OXA–__2_*
27k	*fosA*	*oqxA*	*catA1*	*aac(3), aadA1*	*sul1*	*dfrA*		*bla_SHV–28_, bla_TEM–1B_*
28k	*fosA*	*oqxA*		*strA, strB*				*bla_SHV–__11_*
30k	*fosA*	*oqxA*	*catA1*	*aac(6′), aadA1*	*sul1*			*bla_OXA–2_, bla_TEM–1B_*
31k	*fosA*	*oqxA*		*strA, strB*				*bla_SHV–__11_*
34k	*fosA*	*oqxA*	*catA1*	*aac(6′), aadA1, aadA5*	*sul1*			*bla_OXA–2_, bla_SHV–129_, bla_TEM–1B_*
35k	*fosA*	*oqxA*	*catA1*	*aac(6′), aadA1*	*sul1*			*bla_OXA–2_, bla_TEM–1B_*
36k	*fosA*	*oqxA*	*catA1*	*aac(6′), aadA1, aadA5*	*sul1*	*dfrA*	*tet(A)*	*bla_OXA–2_, bla_TEM–1B_*
37k	*fosA*	*oqxA*	*catA1*	*aac(6′), aadA1, aadA5*	*sul1*	*dfrA*	*tet(A)*	*bla_OXA–2_, bla_TEM–1B_*
40k	*fosA*	*oqxA*						*bla_SHV–11_*
42k	*fosA*	*oqxA*	*catA1*	*aac(6′), aadA1, aadA1*	*sul1*			*bla_OXA–2_, bla_TEM–1B_*
43k	*fosA*	*oqxA*	*catA1*	*aac(6′), aadA1, aadA5*	*sul1*	*dfrA*	*tet(A)*	*bla_OXA–2_, bla_TEM–1B_*
45k	*fosA*	*oqxA*		*aadA1*	*sul1*			*bla_SHV–11_*
46k	*fosA*	*oqxA*						*bla_SHV–1_*
48k	*fosA*	*oqxA*	*catA1*	*strA, strB*	*sul2*		*tet(A)*	*bla_SHV–11_, bla_TEM–1B_*
49k	*fosA*	*oqxA*	*catA1*	*aac(6′), aadA1, aadA5*	*sul1*			*bla_OXA–2_, bla_SHV–129_, bla_TEM–1B_*
50k	*fosA*	*oqxA*	*catA1*	*aac(6′), aadA1, aadA5*	*sul1*			*bla_OXA–2_, bla_TEM–1B_*
**Positives**	**38**	**37**	**19**	**30**	**26**	**15**	**17**	**38**
**Prevalence**	**1.00**	**0.97**	**0.50**	**0.79**	**0.68**	**0.39**	**0.45**	**1.00**

**TABLE 6:** MIC (μg/mL) of the 38 clinical isolates of *K. pneumoniae* according to CLSI.

**Strain**	**FM**	**CF**	**MF**	**CM**	**AK**	**GM**	**TM**	**T/S**	**TC**
2K	>256^R^	>1.0^R^	>1.0^R^	<8.0	>64^R^	>16^R^	>16^R^	>4.0^R^	>16^R^
4K	>256^R^	>1.0^R^	>1.0^R^	>32^R^	>64^R^	>16^R^	>16^R^	>4.0^R^	>16^R^
5K	>256^R^	>1.0^R^	>1.0^R^	>32^R^	>64^R^	>16^R^	>16^R^	>4.0^R^	>16^R^
6K	>256^R^	>1.0^R^	>1.0^R^	>32^R^	>64^R^	>16^R^	>16^R^	>4.0^R^	>16^R^
7K	>256^R^	>1.0^R^	>1.0^R^	<8.0	>64^R^	>16^R^	>16^R^	<2.0	<4.0
8K	>256^R^	>1.0^R^	>1.0^R^	<8.0	>64^R^	>16^R^	>16^R^	<2.0	<4.0
9K	>256^R^	>1.0^R^	>1.0^R^	>32^R^	>64^R^	>16^R^	>16^R^	>4.0^R^	>16^R^
10K	>256^R^	>1.0^R^	>1.0^R^	>32^R^	>64^R^	>16^R^	>16^R^	>4.0^R^	>16^R^
11K	>256^R^	>1.0^R^	>1.0^R^	<8.0	>64^R^	>16^R^	>16^R^	<2.0	<4.0
12K	>256^R^	>1.0^R^	>1.0^R^	<8.0	<16	<4.0	<4.0	<2.0	<4.0
13K	>256^R^	>1.0^R^	>1.0^R^	>32^R^	>64^R^	>16^R^	>16^R^	>4.0^R^	>16^R^
15K	>256^R^	>1.0^R^	>1.0^R^	<8.0	<16	<4.0	<4.0	<2.0	<4.0
16K	>256^R^	>1.0^R^	>1.0^R^	<8.0	<16	<4.0	<4.0	<2.0	<4.0
17K	>256^R^	>1.0^R^	>1.0^R^	<8.0	<16	<4.0	<4.0	<2.0	<4.0
18K	>256^R^	<0.25	<0.25	<8.0	<16	<4.0	<4.0	<2.0	<4.0
19K	>256^R^	>1.0^R^	>1.0^R^	>32^R^	>64^R^	>16^R^	>16^R^	>4.0^R^	>16^R^
20K	>256^R^	>1.0^R^	>1.0^R^	>32^R^	>64^R^	>16^R^	>16^R^	>4.0^R^	>16^R^
21K	>256^R^	>1.0^R^	>1.0^R^	<8.0	>64^R^	>16^R^	>16^R^	>4.0^R^	>16^R^
22K	>256^R^	>1.0^R^	>1.0^R^	<8.0	>64^R^	>16^R^	>16^R^	>4.0^R^	>16^R^
23K	>256^R^	>1.0^R^	>1.0^R^	<8.0	<16	<4.0	<4.0	<2.0	>16^R^
24K	>256^R^	>1.0^R^	>1.0^R^	<8.0	>64^R^	>16^R^	>16^R^	<2.0	>16^R^
25K	>256^R^	>1.0^R^	>1.0^R^	<8.0	>64^R^	>16^R^	<4.0	<2.0	<4.0
27K	>256^R^	>1.0^R^	>1.0^R^	>32^R^	>64^R^	>16^R^	>16^R^	>4.0^R^	<4.0
28K	>256^R^	>1.0^R^	>1.0^R^	<8.0	>64^R^	>16^R^	<4.0	<2.0	<4.0
30K	>256^R^	>1.0^R^	>1.0^R^	>32^R^	>64^R^	>16^R^	<4.0	<2.0	<4.0
31K	>256^R^	>1.0^R^	>1.0^R^	<8.0	>64^R^	>16^R^	<4.0	<2.0	<4.0
34K	>256^R^	>1.0^R^	>1.0^R^	>32^R^	>64^R^	>16^R^	<4.0	<2.0	<4.0
35K	>256^R^	>1.0^R^	>1.0^R^	>32^R^	>64^R^	>16^R^	<4.0	<2.0	<4.0
36K	>256^R^	>1.0^R^	>1.0^R^	>32^R^	>64^R^	>16^R^	>16^R^	>4.0^R^	>16^R^
37K	>256^R^	>1.0^R^	>1.0^R^	>32^R^	>64^R^	>16^R^	>16^R^	>4.0^R^	>16^R^
40K	>256^R^	>1.0^R^	>1.0^R^	<8.0	<16	<4.0	<4.0	<2.0	<4.0
42K	>256^R^	>1.0^R^	>1.0^R^	>32^R^	>64^R^	>16^R^	<4.0	<2.0	<4.0
43K	>256^R^	>1.0^R^	>1.0^R^	>32^R^	>64^R^	>16^R^	>16^R^	>4.0^R^	>16^R^
45K	>256^R^	>1.0^R^	>1.0^R^	<8.0	>64^R^	>16^R^	<4.0	<2.0	<4.0
46K	>256^R^	>1.0^R^	>1.0^R^	<8.0	<16	<4.0	<4.0	<2.0	<4.0
48K	>256^R^	>1.0^R^	>1.0^R^	>32^R^	>64^R^	>16^R^	<4.0	<2.0	>16^R^
49K	>256^R^	>1.0^R^	>1.0^R^	>32^R^	>64^R^	>16^R^	<4.0	<2.0	<4.0
50K	>256^R^	>1.0^R^	>1.0^R^	>32^R^	>64^R^	>16^R^	<4.0	<2.0	<4.0

*MIC, minimum inhibitory concentration; CLSI, Clinical and Laboratory Standards Institute; FM, fosfomycin. Quinolones: CF, ciprofloxacin; MF, moxifloxacin. Phenicols: CM, chloramphenicol. Aminoglycosides: AK, amikacin; GM, gentamicin; TM, tobramycin. Trimethoprim/Sulphonamides: T/S, trimethoprim/sulfamethoxazole. Tetracyclines: TC, tetracycline. R, resistant.*

### The *K. pneumoniae* Genomes Revealed a Myriad of Virulence Factors

**FIGURE 2:**
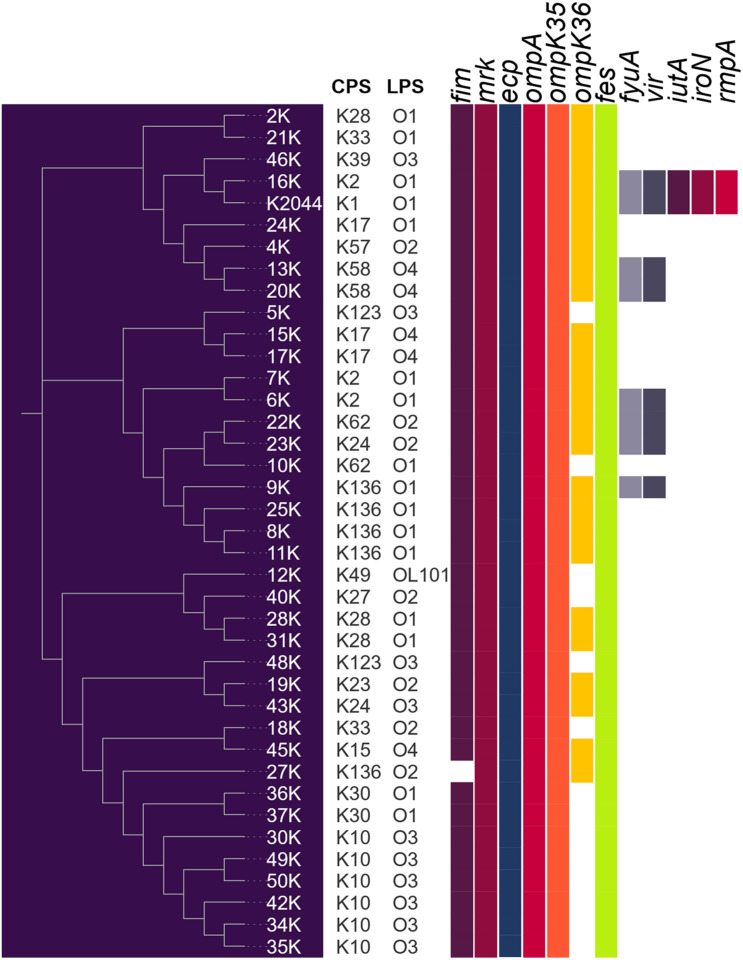
Virulome of *K. pneumoniae* strains. Color filled box mean presence of virulence factor. NTUH-K2044 was added as reference strain.

The prevalence of virulence genes in the genomes of *K. pneumoniae* strains is shown in Figure 2. For fimbrial genes, the prevalence was very high showing that, with exception of strain 27k that lacks the *fim* cluster, all the strains possess the *fim* (Type 1 fimbriae), *mrk* (Type 3 fimbriae) and *ecp* (*E. coli* common pilus) genes. In terms of genes that code for outer membrane proteins, *ompK35* and *ompA* were present in all *K. pneumoniae* genomes, while *ompK36* gene presented in 63% of the strains. Interestingly, seven (18%) *K. pneumoniae* strains were positive for *vir* genes, which code for a putative T4SS. Regarding siderophores, *fes* (enterobactin) and *fyuA* (yersiniabactin) genes were present in 100 and 18% of the *K. pneumoniae* strains, respectively. Of note, only strain 16k carried genes that code for the four siderophores: enterobactin, yersiniabactin, aerobactin, and salmochelin. In addition, this strain was the only one to possess the hypermucoviscous associated gene *rmpA*. None of the strains were positive for *magA* gene. Furthermore, a correlation is depicted between the presence of virulence factors and the clustering by the VGF in Figure 2. Strains 16k and the NTUH K-2044, which possess the higher number of the virulence factors, were grouped into a common clade. Strains 6k, 22k, 23k, and 9k that contain the presence of *fyuA* and *vir* genes, formed an independent clade. Interestingly, strains 13k and 20k that belong to the O4:K58 serotype were allocated into a different clade although they shared the *fyuA* and *vir* genes.

### The *K. pneumoniae* K2 Serotype Is More Resistant to Phagocytosis and Polymyxin B

**FIGURE 3:**
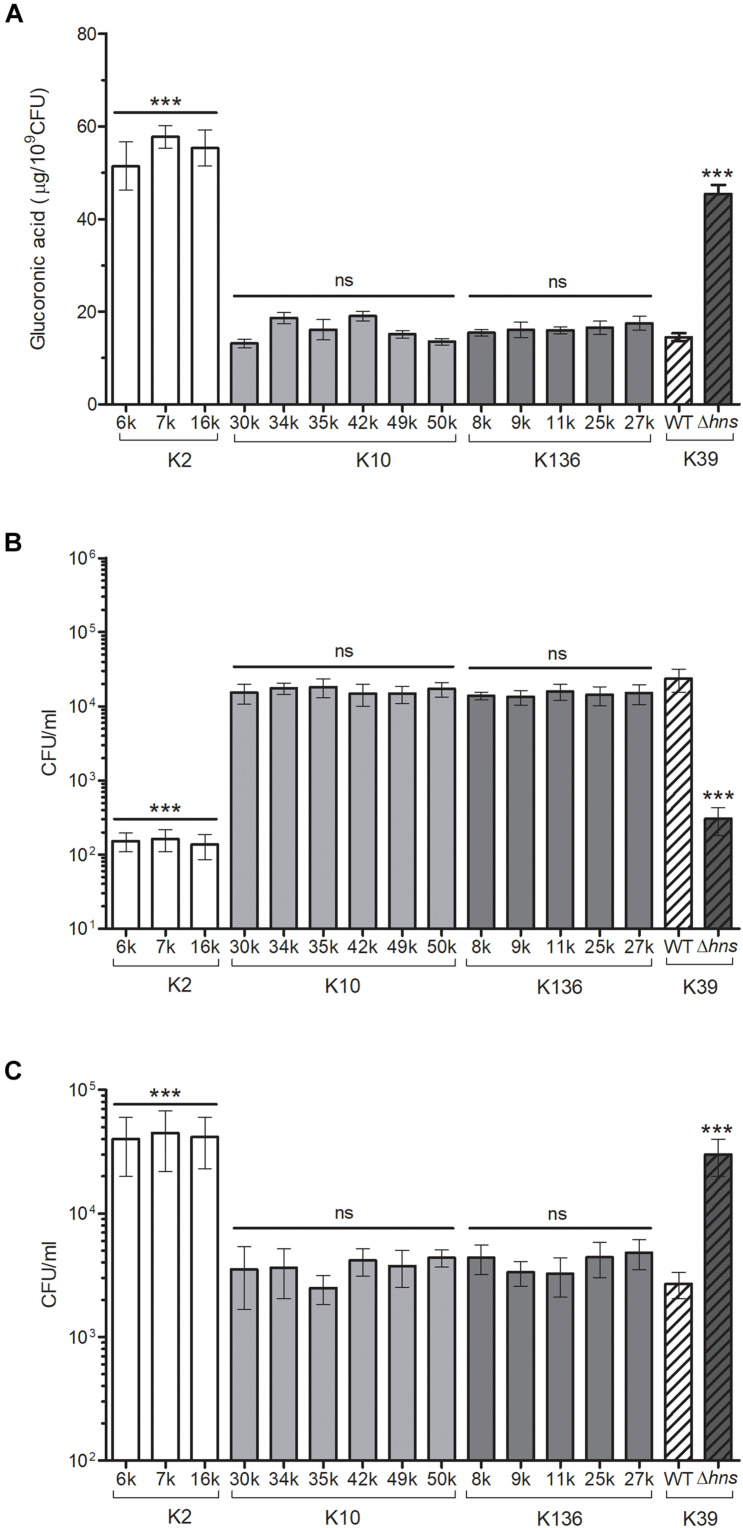
K2 serotype *K. pneumoniae* is highly resistant to phagocytosis and polymyxin B. **(A)** Capsule quantification by determination of the glucuronic acid concentration from capsular polysaccharides extracted from 0.5 ml bacterial cultures. **(B)** Comparison of phagocytic uptake of *K. pneumoniae* grown under the indicated different conditions by THP-1 macrophages. **(C)** Polymyxin B activity (4 mg/ml) against *K. pneumoniae* strains. An Δ*hns* mutant was evaluated as control of phagocytosis and polymyxin B. ns, not significant; statistically significant with respect to bacteria grown in LB medium: ***p < 0.001.

The analysis of the O groups among the strains showed that the most prevalent serogroup was O1 (39%), followed by O3 (26%) (Table 4). Seventeen different K-serotypes were identified in the all strains, being serotype K10 the most prevalent (16%), followed by K136 (13%) (Table 4). Three strains were positive for serotype K2 (6k, 7k, and 16k), which is associated to hypervirulence phenotype due to capsule hyperproduction (Yeh et al., 2007; Yu et al., 2008). We analyzed the capsule production and phagocytosis resistance in *K. pneumoniae* K2 serotype strains and compared them to the K10 and K136 serotypes, which were the most prevalent (Figure 3). In addition, polymyxin B susceptibility assays were performed to evaluate the protective capacity of the capsule against this antimicrobial cationic peptide (Campos et al., 2004). As controls, we used the wild-type *K. pneumoniae* 123/01 strain (K39 serotype) and an Δ*hns*-derivative mutant, which show low and high level of capsule production, respectively (Ares et al., 2016). Indeed, the K2 serotype strains produced more capsule than K10/K136 serotypes to similar levels as the Δ*hns* mutant, which is a hypercapsulated *K. pneumoniae*. Therefore, *K. pneumoniae* K2 serotype strains were less phagocytized by THP-1 macrophages than K10 and K136 serotypes strains. In addition, the action of polymyxin B was lower in the three K2 serotype *K. pneumoniae*.

## Discussion

Gram-negative bacilli infections are the most serious and potentially life-threatening causes of infectious disease in hospitalized patients (Joram et al., 2012; Ares et al., 2013). *K. pneumoniae* is an opportunistic pathogen associated with both community-acquired and nosocomial infections, causing pneumonia, abscess, bacteremia, and urinary tract infections (Gassama-Sow et al., 2010). Due to its ability to rapidly acquire antimicrobial resistance, *K. pneumoniae* has become a global concern calling for actions to prevent the spread of multidrug resistant bacteria (Jansen et al., 2018). Over the last few years, whole-genome sequencing (WGS) has become more accessible and affordable, resulting in its increased use in many fields, including clinical microbiology (Mathers et al., 2015). One of the main benefits of using WGS is to characterize the genomic content of clinically relevant bacteria and relate it to virulence-associated phenotypes to allow the understanding of their transmission within the hospital and to provide accurate diagnostics and to apply prompt therapies. The present study aimed to characterize *K. pneumoniae* isolates from pediatric patients hospitalized between 2010 and 2013 in Mexico City. This is the first study in Mexico that uses WGS to deeply characterize this opportunistic pathogen. In this work, 24 STs were identified in the bacterial genomes analyzed, showing in general, a heterogeneous distribution among which ST-76 (16%) and ST-70 (11%) were the most prevalent STs. Of note, the ST-29 represented by strains 36k and 37k, has previously been associated with hypervirulence (Shon et al., 2013; Lev et al., 2018). This same ST-29 was previously reported in isolates from community-acquired infections in the State of Guerrero, México, presenting a hypermucoviscosity phenotype (Garza-Ramos et al., 2018).

Regarding the prevalence of antibiotic resistance, *K. pneumoniae* genomes showed high prevalence (97%) for *oqxA* or *qnrB* genes, which conferred resistance to quinolones. Only one strain (18k) was sensitive to ciprofloxacin and moxifloxacin, which corresponded to the lack of *oqxA*/*qnrB* genes in the bacterial genome. This high resistance to this class of antibiotics has been reported by other researchers in both Mexico and worldwide (Rodríguez-Zulueta et al., 2013; Osei Sekyere and Amoako, 2017; Salah et al., 2019; Geetha et al., 2020). Sulfonamide resistance genes such as *sul1* or *sul2* were highly prevalent (68%) in the *K. pneumoniae* strains analyzed in this study, which is in agree with other reports (Khamesipour and Tajbakhsh, 2016; Taitt et al., 2017). Genes that confer tetracycline [*tet(A), tet(B), tet(C), tet(D), or tet(E)*] were present in 45% of the strains, which is low in comparison to other studies (Bokaeian et al., 2015; Lev et al., 2018). All strains presented at least one type of gene coding for β-lactamases and the SHV-type was the most prevalent in the sequenced genomes (53%). However, for the *bla*_TEM_-type, only *bla*_TEM–1B_ gene was identified in 20 strains (53%), which has been reported as the most prevalent β-lactamases genes in *K. pneumoniae* (Chang et al., 2001; Feizabadi et al., 2010; Shaikh et al., 2015). The β-lactamases genes *bla_OXA–1_ and bla_OXA–2_* were found in 20 strains (53%), while for the *bla*_CTX–M–15_ gene was found in 10 bacterial genomes analyzed (26%), which has been reported by different authors as common in nosocomial outbreaks (Zhou et al., 2015; Bevan et al., 2017; Gautam et al., 2019). β-lactamases genes *bla*_OKP_ and *bla*_LEN_ were not found in the strains.

Seventeen different types of CPS were found among the clinical *K. pneumoniae* collection. Three strains (6k, 7k, and 16k) were identified as belonging to the K2 capsular serotype, which is the main antigen associated with hypervirulence in *K. pneumoniae* (Brisse et al., 2013; Lin et al., 2014; Lev et al., 2018). These three strains produced more capsule and consequently they were more resistant to both macrophage-mediated phagocytosis and polymyxin, than the K10 and K136 capsular types, which were the most prevalent serotypes in our study. Our results are consistent with previous studies confirming that the K2 serotype confer *K. pneumoniae* higher resistance to both macrophage-mediated phagocytosis and polymyxin B due to increased capsule production (Yu et al., 2015). In the case of the LPS O-antigen, five different serotypes were found. In agreement with other studies (Shankar-Sinha et al., 2004; Hsieh et al., 2014; Follador et al., 2016), the O1 antigen, which confers greater resistance to the serum’s bactericidal activity, was the most prevalent O1 serogroup (39%) in the *K. pneumoniae* strains isolated from blood. Whilst O2 serogroup is more sensitive to human serum killing than O1 (Pennini et al., 2017), there are not reports about the O3 antigen immunogenicity, which was the second serogroup most prevalent (24%) in the *K. pneumoniae* strains collection.

In terms of adhesive structures, type 3 fimbria is the most important organelle of *K. pneumoniae*, playing an important role in adherence to both abiotic and biotic surfaces (Langstraat et al., 2001; Di Martino et al., 2003; Struve et al., 2009; Schroll et al., 2010; Ares et al., 2016). Virulence gene analysis revealed that the *mrk* cluster was present in all the strains, supporting the notion that type 3 fimbriae are a hallmark of *K. pneumoniae* virulence. Porins have been shown to be important for bacterial survival due to their role in the exchange of substances, including nutrients and toxic metabolites (Tsai et al., 2011). All the strains presented both the *ompA* and *ompK35* genes. In contrast, only 25 (66%) *K. pneumoniae* strains were positive for *ompK36* gene. The high prevalence of *ompK35 versus ompK36*, supports the structural role of OmpK35 as a porin related to the outer membrane integrity, whilst OmpK36 provides increased antibiotic resistance with no significant loss of fitness in the host (Fajardo-Lubián et al., 2019). Interestingly, *K. pneumoniae* strains lacking *ompK36* presented over 70% prevalence of *bla*_OXA_ carbapenemase gene, which support the notion that the resistance to carbapenem antibiotics is related to the absence of *ompK36* porin gene (Landman et al., 2009; Wong et al., 2019). The combination of siderophores secreted by *K. pneumoniae* during infection affect tissue localization, systemic dissemination, and host survival (Holden et al., 2016). Enterobactin genes were present in 100% of the *K. pneumoniae* genomes, which is understandable since the catecholate is typically encoded in the core genome of *K. pneumoniae* (Martin and Bachman, 2018). Genes that encodes yersiniabactin siderophore were present in seven (18%) *K. pneumoniae*, and two of these genomes presented the K2 capsular serotype, reflecting the virulence properties of these strains, since yersiniabactin is significantly associated with invasive infections in humans (Holt et al., 2015).

With exception of the strain 46k, at least one plasmid sequence was detected in 37 of the 38 genomes analyzed. IncFIB plasmid was present in 36 of 37 of the bacterial isolates. This conjugative plasmid has been associated with the dissemination of the *bla_NDM–1_*, *bla_SHV–12_*, *bla_CTXM–15_*, and *bla_OXA–1_* genes in *K. pneumoniae* (Oliveira et al., 2020). Methods of circularization and annotation of the plasmid DNA using sequencing techniques that generate long-read sequences will offer insights into the content of those plasmids and its evolution and spread (Ramsamy et al., 2020).

Traditional phylogenetic approaches require the calculation of multiple sequence alignment of homologous sequences for finding informative characters or for the calculation of sequence differences that are used as overall measures to estimate evolutionary distances. Thus, the time required for a phylogenetic study is distributed into that which is needed for calculating the alignment and that which is needed for the phylogenetic method itself (Yang and Rannala, 2012). The VAMPhyRE method is an alignment free tool based in the calculation of Virtual Genomic Fingerprints by a virtual hybridization with predesigned probe sets. Additionally, SNPs methods usually require a collection of SNPs relative to a reference sequence which is not required by the VAMPhyRE approach. The phylogenomic reconstructions obtained by VAMPhyRE correlated to the MLST determination. These results demonstrated a strong relation between MLST classification scheme and the phylogenomic analysis in the bacterial strains. This same behavior has been reported by Muñoz-Ramírez et al. (2017) in *Helicobacter pylori* strains. Furthermore, the VAMPhyRE methodology was able to cluster together strains with high number of virulence factors and hypervirulent serotypes from those lacking them. Interestingly, strain 16k presented a strong phylogenetic relation with NTUH-K2044 reference strain, carrying all virulence factors analyzed in our study, including the four siderophores and the T4SS genes. Ongoing work in our laboratory aims to characterize the pathogenic features of this strain. In summary, the search of virulence factors by using whole genome sequencing and virtual genome fingerprinting is an alternative method to classify clinical *K*. *pneumoniae* strains.

## Data Availability Statement

The datasets generated for this study can be found in online repositories. The names of the repository/repositories and accession number(s) can be found in the article/Supplementary Material.

## Author Contributions

MD and AM-T conceived and designed the experiments. MA and RR-R performed the experiments. MF-V, MA, JT, JG, BW, AM-T, and MD analyzed the data. MF-V, JG, and MD wrote the manuscript. All authors contributed to the article and approved the submitted version.

## Conflict of Interest

The authors declare that the research was conducted in the absence of any commercial or financial relationships that could be construed as a potential conflict of interest.

## Publisher’s Note

All claims expressed in this article are solely those of the authors and do not necessarily represent those of their affiliated organizations, or those of the publisher, the editors and the reviewers. Any product that may be evaluated in this article, or claim that may be made by its manufacturer, is not guaranteed or endorsed by the publisher.

## Footnotes

1
http://biomedbiotec.encb.ipn.mx/VAMPhyRE

2
https://bigsdb.pasteur.fr/klebsiella/klebsiella.html

3
http://www.genomicepidemiology.org

4
https://kaptive-web.erc.monash.edu
