# Supplementary material for: Whole Genome Sequencing of Pediatric Klebsiella pneumoniae Strains Reveals Important Insights Into Their Virulence-Associated Traits
Source: Front Microbiol. 2021 Aug 13;12:711577. doi: 10.3389/fmicb.2021.711577 (PMC8418058; doi:10.3389/fmicb.2021.711577)
Supplement: Supplementary file 1 [file Table_1.DOCX]

**Supplementary table 1.** Locus tags of genes screened.

| **Antibiotic type** | **Gene screened** | **Locus tag** |
| --- | --- | --- |
| Fosfomycin | *fosA* | ABY74375 |
| Quinolones | *oqxA* | AIS72928 |
|  | *qnrB* | ASA69712 |
| Phenicols | *catB3* | APU91754 |
|  | *catA1* | QOQ32345 |
| Aminoglycosides | *strA* | ARA74257 |
|  | *strB* | ARA74258 |
|  | *aac(3)* | AAA98404 |
|  | *aac(6')* | AAA98404 |
|  | *aadA1* | AAA98405 |
|  | *aadA5* | AHK14455 |
| Sulphonamydes | *sul1* | APD70824 |
|  | *sul2* | APD70845 |
| Trimethoprim | *dfrA* | QUR77706 |
| Tetracycline | *tetA* | ABS19074 |
| β-lactamase SHV | *bla*_SHV-1_ | BBW86743 |
|  | *bla*_SHV-11_ | QOQ32770 |
|  | *bla*_SHV-12_ | QOQ32601 |
|  | *bla*_SHV-28_ | BBS42421 |
|  | *bla*_SHV-38_ | ACV33212 |
|  | *bla*_SHV-129_ | ADI87569 |
| β-lactamase OXA | *bla*_OXA-1_ | QOQ32955 |
|  | *bla*_OXA-2_ | SMY31119 |
| β-lactamase TEM | *bla*_TEM-1b_ | ARA74260 |
| β-lactamase CTX | *bla*_CTX-M-15_ | QGW59023 |
| **Structure** | **Gene screened** | **Locus tag** |
| Type I pilus | *fimB* | KP1_RS21310 |
|  | *fimE* | KP1_RS21315 |
|  | *fimA* | KP1_RS21325 |
|  | *fimI* | CDO12577 |
|  | *fimC* | CDO12576 |
|  | *fimD* | CDQ52831 |
|  | *fimF* | CDO12574 |
|  | *fimG* | CDO12573 |
|  | *fimH* | CDO12572 |
|  | *fimK* | ACV60161 |
| Type III pilus | *mrkA* | KP1_RS21280 |
|  | *mrkB* | KP1_RS21275 |
|  | *mrkC* | KP1_RS21270 |
|  | *mrkD* | KP1_RS21265 |
|  | *mrkF* | KP1_RS21260 |
| *E. coli* Common Pilus | *ecpA* | KP1_RS05450 |
|  | *ecpB* | GHS67316 |
|  | *ecpC* | QTZ86585 |
|  | *ecpD* | KP1_RS05465 |
|  | *ecpE* | QTZ86583 |
| Outer Membrane Protein A | *ompA* | KP1_RS09230 |
| Outer Membrane Protein 35 | *ompK35* | KP1_RS09095 |
| Outer Membrane Protein 36 | *ompK36* | APB87317 |
| Type IV Secretion System | *virB1* | QOQ33227 |
|  | *virB2* | AFV66834 |
|  | *virB3* | QGM50301 |
|  | *virB4* | AWD73384 |
|  | *virB5* | AFV66836 |
|  | *virB6* | AFV66838 |
|  | *virB7* | AFV66839 |
|  | *virB8* | AFV66840 |
|  | *virB9* | QBA99769 |
|  | *virB10* | AFV66842 |
|  | *virB11* | AGZ05422 |
| Mucoviscosity-associated gene A | *magA* | VAO81655 |
| Regulator of mucoid phenotype A | *rmpA* | KP1_RS16895 |
| Enterobactin | *fes* | KP1_RS07260 |
| Salmochelin | *iroN* | KP1_RS16865 |
| Yersiniabactin | *fyuA* | KP1_RS16805 |
| Aerobactin | *iutA* | KP1_RS26875 |
